# Variation among *Metschnikowia pulcherrima* Isolates for Genetic Modification and Homologous Recombination

**DOI:** 10.3390/microorganisms9020290

**Published:** 2021-01-31

**Authors:** Mauro Moreno-Beltrán, Deborah Gore-Lloyd, Christopher Chuck, Daniel Henk

**Affiliations:** 1 Milner Centre for Evolution, Department of Biology and Biochemistry, University of Bath, Bath BA2 7AY, UK; M.Moreno@bath.ac.uk (M.M.-B.); D.L.Gore-Lloyd@bath.ac.uk (D.G.-L.); 2 Centre for Integrated Bioprocessing Research, Department of Chemical Engineering, University of Bath, Bath BA2 7AY, UK; C.Chuck@bath.ac.uk

**Keywords:** nonconventional yeasts, biotechnology, homologous recombination

## Abstract

*Metschnikowia pulcherrima* is a non-conventional yeast with the potential to be used in biotechnological processes, especially involving low-cost feedstock exploitation. However, there are a lack of tools for researching it at a molecular level and for producing genetically modified strains. We tested the amenability to genetic modification of ten different strains, establishing a transformation protocol based on LiAc/PEG that allows us to introduce heterologous DNA. Non-homologous integration was broadly successful and homologous recombination was successful in two strains. Chemical inhibition of non-homologous end joining recombination had a modest effect on the improvement of homologous recombination rates. Removal of selective markers via flippase recombinase was successful across integrated loci except for those targeted to the native *URA3* locus, suggesting that the genome sequence or structure alters the efficacy of this system.

## 1. Introduction

Novel yeasts and their natural variants offer exceptional promise for improved industrial biotechnology, from utilization of sustainable feedstocks through to production of high-value compounds [1,2]. Whilst exploiting natural strains is an important strategy, developing genetic tools in these novel yeasts is critical for establishing flexibility and opening the applications for specific yeast products and, importantly, to understand the basic biology of these yeasts [3]. Genetic tools in the model yeast *Saccharomyces cerevisiae* are incredibly powerful, but there are many challenges in establishing even a basic genetic tool kit for a new yeast, as each new yeast has a unique set of molecular and genomic features that require specialised manipulation approaches [4,5,6,7,8]. Arguably, the first main challenges are: (1) getting detectably functional DNA into the yeast; (2) targeting the DNA within the genome; (3) having a system that allows more than one round of modification. These issues are not likely to be monolithic within a species and if a major goal is to exploit natural variation, then it is important to determine how tools differ across strains within a species as well in the novel species.

While it is well recognized that developing genetic tools for a novel species can be challenging, variation within a species is often ignored in discussions around applications within yeasts [3]. Yet, strains within species can differ dramatically in nearly every element that makes genetic manipulation feasible. When *S. cerevisiae* was being developed as a model for genetic manipulation many strains were initially considered and only after wide screening of many strains were the standard strains for future work and optimisation established [9]. Yet much of the potential application of yeast resides in non-optimised strains and it remains an ongoing challenge within *S. cerevisiae* to reliably manipulate relevant natural strains for industry [3]. In novel yeasts, there is usually little known about natural variation in most traits, including how strains differ in their molecular mechanisms that allow manipulation or even in basic genomic features such as ploidy.

In order to genetically manipulate yeast, it is often required to introduce exogenous DNA into them. This means that the DNA needs to traverse the cell wall and cell membrane, avoid digestion within the cytoplasm and enter the nucleus where it can be either maintained in plasmid form or integrated into the genome [10]. This basic transformation process is the first tool needed in order to alter the genome of yeasts for applications in the lab or industry. The most common protocol used to transform *S. cerevisiae* is the LiAc/PEG method which has been optimised many times, reaching a high rate of success [11,12]. However, it is often the case that different species of yeast need modifications of this protocol in order to achieve an efficient process [13,14].

While most DNA integration in yeasts is random with respect to genomic location, targeting a specific gene allows the disruption of its expression and the proteins that it encodes, which can be used to study the function of that gene and to obtain mutants with desirable phenotypes for industry [15]. Newer techniques that can target specific sequences such as CRISPR/Cas9 are incredibly useful in editing genomic DNA, but multiple hurdles persist in developing these techniques in novel yeasts [16]. While the bacterial-derived CRISPR systems offer many opportunities, the native HR (homologous recombination) rates in many yeasts can be sufficient for industrial applications and exploration of metabolism where genomic scarring is not important and marker recycling is feasible. In these systems, the sequence of the targeted locus can be added on the sides of the exogenous DNA introduced, which allows the homologous recombination (HR) machinery to recognize it and swap it with the endogenous DNA, and the use of HR to target loci is a common tool for the manipulation of yeasts. However, HR is a natural metabolic process with the function of replicating and preserving the genome against DNA damages such as DNA gaps, interstrand crosslinks (ICLs) and double-stranded breaks (DSBs). When the damage to the DNA causes DSB, its repair can be carried out by HR or non-homologous end joining (NHEJ). These two pathways establish a balance to repair DSBs which varies for different species or cell cycle phases inside a single cell [17]. In yeast, the relationship between these two pathways is strongly dominated by the NHEJ. *S. cerevisiae* and *S. pombe* as they have higher rates of HR and are exceptions to this norm which makes them easier to work with compared to non-conventional yeasts in which the low gene-targeting efficiency can compromise their research and application [18]. Multiple approaches facilitate increased efficiency of this process, particularly varying the length of homologous stretches of DNA incorporated into the vector [6,19,20].

There are several options to bypass the predilection of yeasts for NHEJ over HR. One of them is to create mutants that are defective in the NHEJ pathway [21]. The most common approach to do this is to eliminate the *ku* genes which encode the DNA-dependent protein kinase heterodimeric regulatory factor *Ku70*-*Ku80*. This complex forms a link between the two damaged DNA ends and recruits other proteins, thereby acting as the starting point of NHEJ [22]. However, the deletion of these genes is not a trivial task given that gene targeting in yeasts with low HR is challenging. Furthermore, deletions may be lethal [23] or create instability in the genome causing increased mutation rates and sensitivity to DNA damage [19,24]. An alternative to the deletion or mutation of the genes responsible for NHEJ is to use chemicals to transiently inhibit the activity of the encoded proteins, an approach that has have been proven to have a big impact on HR rates [25]. Another method which avoids the need to delete the *ku* genes is based on the fluctuation of the NHEJ/HR rates during the different cell phases. HR is the predominant pathway for DSB repair during S/G2 phase, so by synchronizing most of the cell population during transformation with the aid of an inhibitor of ribonucleotide reductase it is possible to promote HR [26]. Both chemical methods to inhibit NHEJ in a transient manner are very useful for non-conventional yeast given that they target highly conserved processes and they do not require to target a gene deletion trough HR.

Although some of the yeast strains that are most often used in research are haploid, ploidy yeasts require extra steps for HR-mediated gene disruption/deletion. In the case of diploid yeasts there are two copies of the gene that have to be targeted via two rounds of transformation, each of which requires a selectable marker. Auxotrophic markers are commonly used for genetic research on yeasts, with the auxotrophic strains being generated either by random mutation or HR itself. Genes that confer resistance to an antibiotic are also used as markers. Regardless of the marker type, two are needed in order to delete two copies of a gene, or the same marker can be used twice if it is recycled. In yeast, marker recycling is commonly done by using the flippase recombinase (FLP) under an inducible promoter thus enabling removal of the marker once the transformant has been selected. FLP recognizes a specific sequence, called FRT, which is placed on both sides of the selectable marker, then FLP circularizes the DNA that is in between the FRT sequences and gets discarded. Once the selection marker is eliminated from the transformant, the second copy of the gene can be targeted using the same plasmid as in the first round [27]. This technology has been applied to several diploid yeasts with success and it is a standard procedure to produce knockout strains [28,29].

*Metschnikowia pulcherrima* is a promising yeast for multiple industrial applications, particularly in the areas of sustainable exploitation of low-cost feedstocks [30,31]. The species is part of a complex of species within the CUG alternative codon using clade that includes multiple pathogenic and industrial yeasts [2,32]. Its natural ecology is complex including many different habitats, heterothallic mating structure and rapid diversification [2,33,34]. Much of the attention for *M. pulcherrima* has come from its recently recognized oleaginous status, accumulating lipids up to 40% of its dry weight, and the flexibility of its metabolism which can use a wide range of carbon and nitrogen sources [31,35]. Antimicrobial properties of *M. pulcherrima* and its close relatives have already attracted considerable attention including applications in biocontrol and research directly to discover the underlying antimicrobial molecular mechanisms [36,37,38,39]. In the winemaking industry, *M. pulcherrima* has broad use in producing enhanced flavour profiles particularly in lower alcohol products [40,41]. In recent work focused on sustainable and circular chemical technologies, *M. pulcherrima* showed flexibility and applicability at scale in producing 2-phenylethanol and lipids from both complex substrates such as algae and from standard feedstocks such as starch [42,43,44].

Despite its potential value in industry from agriculture to fuels, relatively little is known about genetic manipulation in this yeast, but there is strong evidence for important differences between strains in their applicability [34,35,45,46,47]. Here, we seek to establish some of the basic tools for genetic manipulation of *M. pulcherrima* and assess the variation in tractability between strains within this species.

## 2. Materials and Methods

### 2.1. Strains and Media

*M. pulcherrima* strains from our collection (Table 1) were cultured in SMB (30 g/L tryptic soy broth (Sigma, St. Louis, MO, USA), 25 g/L malt extract (pH5, Sigma, St. Louis, MO, USA) or malt extract agar (MEA, Sigma, St. Louis, MO, USA) at 25 °C. Nourseothricin (Nat, Werner BioAgents, Jena, Germany, 50 µg/mL) was used to select transformants.

### 2.2. Generation of the URA3 Deletion Construct

**Figure 1:**
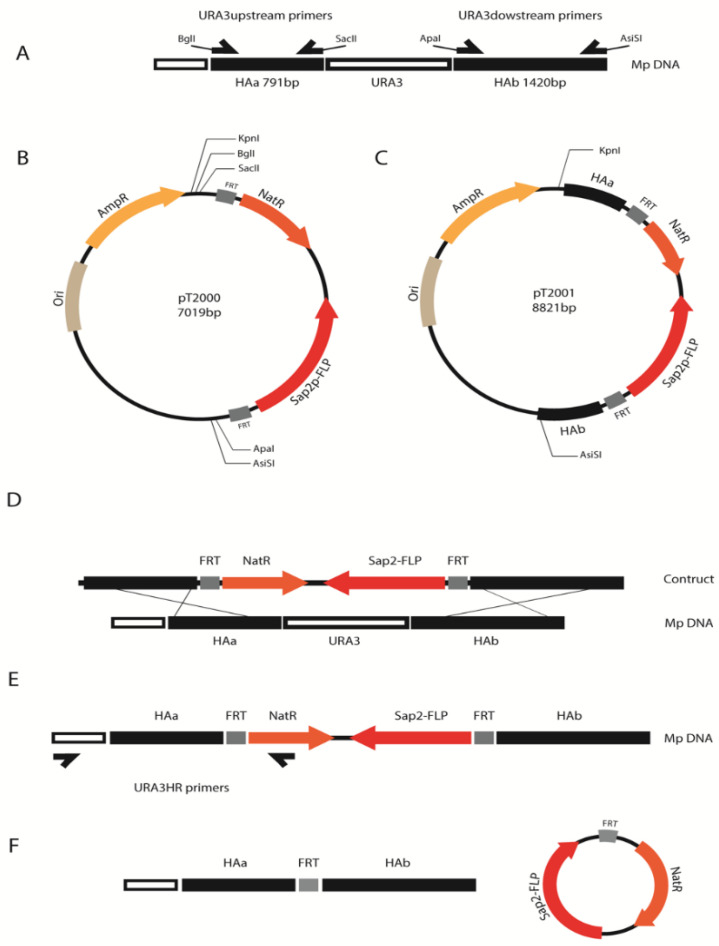
Diagram of the cloning process, homologous recombination and flippase recombinase activity. (**A**) Homologous arms (HAa and HAb) were amplified from *M. pulcherrima* by PCR using the pairs of primers URA3upstream and URA3downstream which have a restriction enzyme recognition site on their 5′. (**B**) pT2000 plasmid which contains an origin of replication for *E. coli* (Ori), an ampicillin resistance gene (AmpR), the NatR (nourseothricin) and FLP (flippase recombinase) genes between FRT sequences and some single cut restriction enzyme recognition sites outside the FRTs. (**C**) Both homologous arms were cloned into pT2000 by sequential enzyme restriction, ligation and propagation on E. coli, generating the plasmid pT2001. This plasmid was linearized with KpnI and AsiSI prior to being transformed into *M. pulcherrima*. (**D**) Homology recognition takes place leading to the replacement of the *URA3* gene for the NatR-FLP construct. (**E**) Homologous recombination screening by PCR using a primer that binds to the NatR gene and another primer that anneals to a sequence in the genome immediately adjacent to one of the homology arms. (**F**) Result after the activation of the FLP recombinase by the induction of the Sap2 promoter, which leads to discard of NatR and therefore the ability to grow in Nat-supplemented media.

To create the *URA3* deletion construct, homology arms 791 kb upstream and 1420 kb downstream of the *URA3* gene locus in *M. pulcherrima* were amplified by PCR with oligos pairs URA3upstream FW/RV and URA3downstream FW/RV, respectively (Table 2). Restriction enzyme sites were added to the end of the homology arms during the PCR to enable ligation into a pT2000 plasmid backbone via BglII/SacII and ApaI/AsiSI (New England Biolabs, Ipswich, MA, USA), resulting in the plasmid pT2001 (Figure 1). The plasmid was propagated in competent *E. coli* (5-alpha, New England Biolabs, Ipswich, MA, USA) and cultured in Luria broth (10 g/L NaCl, tryptone 10 g/L, yeast extract, 5 g/L, Sigma, St. Louis, MO, USA) with 100 μg/mL ampicillin (Sigma, St. Louis, MO, USA). In between the homology arms, the plasmid contains a NAT-FLP cassette comprised of a *Candida albicans* codon-optimized selection marker nourseothricin (Nat^R^) and an inducible promoter SAP2 (from *C. albicans*) controlling the expression of FLP. Flanking the NAT-FLP cassette are two FRT sites for selection marker recycling via recombination upon induction of the FLP gene. Plasmids were digested with KpnI/AsiSI (New England Biolabs, Ipswich, MA, USA) prior to transforming *M. pulcherrima*.

### 2.3. Yeast Transformation

*M. pulcherrima* was transformed with a modified protocol from [11]. Overnight cultures were diluted to an OD_600_ = 0.3 and grown until they reach OD_600_ = 0.8–1. 1 mL of culture per transformation was pelleted, washed once with PBS (Oxoid, Leicester, UK), then were resuspended in 260 µL of transformation mix 4 µg of linearized DNA, 100 µL 10 × tris-EDTA (Fisher Scientific, Loughborough, UK), 100 µL 1 M lithium acetate (Sigma, St. Louis, MO, USA) pH 7.4, 40 µL 5 mg/mL salmon sperm DNA (AppliChem, Darmstadt, Germany) previously boiled, 20 µL 1 M DTT (Sigma, St. Louis, MO, USA), then 800 µL of polietinelglicol (PEG, Sigma, St. Louis, MO, USA) 3350 was added. Transformations were incubated at 25 °C overnight. The cells were heat shocked at 40 °C for 5 min then placed on ice for 1 min. After centrifugation for 5 min at 1100 g, the supernatant was removed and the cells were resuspended in SMB, then incubated for 2 h at 25 °C, 200 rpm, then plated on to MEA containing 50 μg/mL Nat and placed in a static incubator at 25 °C for 2–3 days until colonies appeared.

### 2.4. Transformants Screening

Colonies that grew on transformation plates were patched onto fresh MEA + Nat plates to confirm antibiotic resistance. PCR was used to look for targeted genomic integration of the deletion construct (Table 2). A small amount of cells from the patches were diluted in water without pretreatment and subjected to whole-cell PCR with DreamTaq Green (ThermoFisher, Oslo, Norway) and primers NatHRScreen (Table 2). Primer NatHRScreen-RV annealed to a sequence within the Nat gene whilst primer 5977HRScreen-FW annealed to a sequence in the genome immediately adjacent to one of the homology arms (Figure 1) [26].

### 2.5. Heat Shock Evaluation

To test the viability of cells after heat shock, each overnight culture was diluted to an OD_600_ = 0.3 and grown until they reached OD_600_ = 0.8–1.1 mL of each culture was heat shocked at 40 °C for 5 min, then placed on ice for 1 min. Cell counting was performed using FastRead counting slides (Inmune Systems, Torquay, UK). Dilutions were performed to get 100 cells of each strain to plate them into MEA. Colony number was counted to calculate the survival of the strain after the heat shock [50].

### 2.6. Drug Treatments to Increase HR

Two drugs that inhibit NHEJ, N-(6-aminohexyl)-5-chloro-1-naphthalenesulfonamide (W7, Tokyo Chemical Industry, Tokyo, Japan) [25] and hydroxyurea (HU, Sigma, St. Louis, MO, USA) [26], were used prior to transformation. After diluting the overnights to OD_600_ = 0.3, all the strains were grown for two hours before adding these drugs to the media and were then incubated for three hours before pelleting them and following with the transformation protocol.

It was determined that 60µg/mL of W7 and higher concentrations reduce the viability of the *M. pulcherrima* cultures thus W7 was tested at the following concentrations: 7.5, 15 and 30 µg/mL. Different concentrations of HU were evaluated and the lowest concentration which arrested around 90% of the population (determined by use of the microscope) in two hours was 25 mM.

### 2.7. Selection Marker Recycling

In order to activate the FLP recombinase expression, which is regulated by the inducible SAP2 promoter, *M. pulcherrima* was cultured at 25 °C and 200 rpm in yeast nitrogen base without amino acids and ammonium sulphate (1.7 g/L, Sigma, St. Louis, MO, USA) with 4 g/L bovine serum albumin (BSA, Sigma, St. Louis, MO, USA), 2 g/L of yeast extract and 20 g/L of maltose (Sigma, St. Louis, MO, USA) [28]. After three days a dilution 1:1,400,000 was performed and 150 µL was plated in MAE without Nat. Once the colonies were grown the plates were replicated in MEA and MEA with Nat.

## 3. Results

### 3.1. Strain Level Variation in HR and Response to Heat Shock

We used ten strains of M. pulcherrima that varied in several traits thought to be relevant in industry and natural ecology (Table 1). These strains represent natural variation and capture a range of potential applications including their amenability to genetic modification. Among the strains tested, we observed HR in only two and the frequency of HR was very low (Table 3). Eight of the transformed strains showed over 100 colonies when first grown on Nat-containing media, yet most colonies did not survive when they were transferred and regrown in another round of Nat media, suggesting that it is likely that constructs were not integrated into the genome effectively and were lost upon propagation or that Nat genes were poorly or unstably expressed. The percentage of stable colonies as a function of the total initial Nat resistance colonies varied between 8–36%. Similarly, the effect of heat shock differed considerably between strains with 11.7–63.4% of cells surviving. Strain ICS48 had the highest survivability, but showed the lowest frequency of cassette integration while the strain NCYC3047 had the lowest survivability but was among the two strains with successful HR. Taken together, these results show that when all strains are treated with similar protocols they have strikingly different responses in their genetic tractability.

### 3.2. Chemical Suppression of NHEJ in a Strain with Low but Detectable HR

We tried to improve the HR via chemical suppression of NHEJ. To do this we used strain NCYC2580 because it has a detectable frequency of HR and has a significant number of successful transformants that are not integrated at the target locus, suggesting the best likelihood for improvement. Applying the NHEJ-inhibiting drugs HU and W7 to the transformation protocol resulted in a modest increase in the frequency of HR transformation. We found that among those tested, only the HU 25 mM and W7 30 µg/mL drugs resulted in any detectable HR strains (4% and 2% respectively, out of 50 colonies). In the lower doses of W7 and the no treatment control there were no detectable transformants, consistent with lower efficiency at lower chemical concentration.

### 3.3. Marker Recycling via FLP in a Diploid Strain

**Figure 2:**
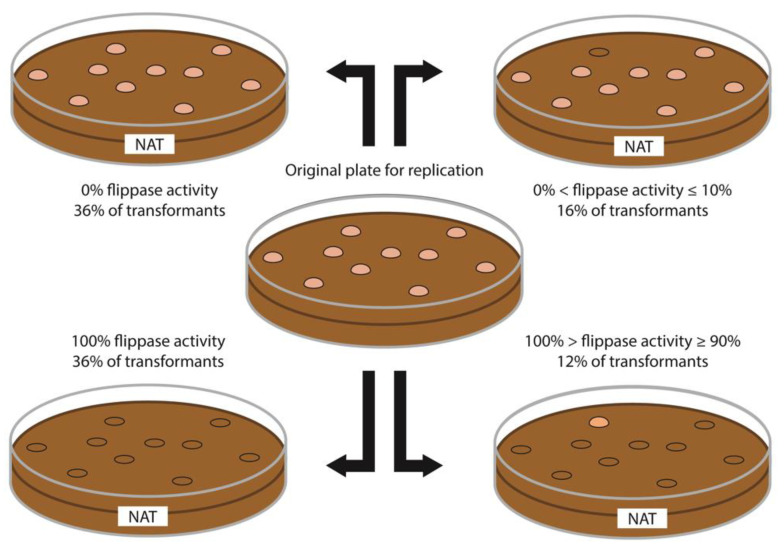
Representation of the plate replication results after activation of the FLP recombinase. Use of Nourseothricin on a plate is represented with the text NAT on the diagram. A total of 25 transformants of the strain NYCY3047 were examined after FLP recombinase activation showing a FLP activity either equal to or higher than 90% activity or equal to or lower than 10%. Most of the transformants (72%) showed activity in all or none of the colonies, while the rest had either very high or low FLP activity. These results confirm the efficacy of the SAP2 promoter/FLP recombinase system on M. pulcherrima even though none of the HR transformants show any FLP activity, therefore impeding a second round of transformation with the same construct.

We aimed to use a common marker recycling system for diploid yeasts. Flanking the selectable marker with FRT sequences allows for its excision by FLP recombinase and thus for its use in a second round of transformation. Using NCYC2580, the strain that was most successfully modified via HR to generate a deleted *URA3* gene, we sought to develop a *Ura3*Δ diploid strain by recycling the Nat selection marker with aid of the FLP system under inducible expression via the SAP2 promoter. The function of FLP under SAP2 control was confirmed by examination of 25 transformants of the diploid strain NCYC3047 (Table 3). However, among the colonies derived from NCYC2580 at which HR was detected, no FLP activity was detected. Among all the tested transformants, the FLP activity of each was either equal or higher than 90% or equal or lower than 10%. The majority of the tested transformants (72%) showed resistance to Nat in all or none of the colonies (Figure 2). Therefore, the activity of the system varies based on the specific transformant and can either essentially work for any replicate or fail for any replicate, but few transformants show intermediate efficacy of the FLP system across replicates. These results confirm the efficacy of the inducible SAP2 promoter and demonstrate that the FLP system can function with this integrated sequence, but critically for HR tools, none of the transformants with the construct at the native *URA3* locus were among those with functional FLP systems.

## 4. Discussion

Developing standard tools for novel microbes is a central goal in the development of the new industrial biotechnology pipeline and part and parcel of this strategy is understanding the basic biology of the microbial system being targeted. Some of the principal challenges include mitigating or capitalizing on variation between strains in their suitability for each specific method of genetic modification and detailing the specific mechanisms of genomic repair and recombination, namely NHEJ and HR, that affect the performance of these tools.

Even though strain-level variation in HR has been described in other yeasts [51,52], *M. pulcherrima* shows more variable responses that make prediction of any particular method of transformation and HR based on heat shock or other culture condition unlikely to be accurate for more than one strain. There is a not an obvious pattern in effects between strains, but the strains are clearly different in tractability and HR is likely to be of only limited use given the current system, but there are at least some candidate strains to use for HR in *M. pulcherrima*. These strains can be used to explore gene deletions that can improve phenotypes with industrial interest, such as lipid accumulation [53].

Other yeasts have shown much more substantial increases in efficiency using the drugs we tested for suppression of NHEJ [25,50], but these yeasts are mostly already showing higher rates of HR than we detected in *M. pulcherrima*. The relative increase in efficiency we observed is similar to that found in *Yarrowia lipolytica* targeting YALI0D17534, but this level of HR was found to be site dependent [26]. Given these results, recurring to deleting NHEJ-related genes may be another route to be taken in order to improve the HR rates [22].

Because the primary difference between the transformants is in the insertion site of the marker, we hypothesize that the genome structure itself dictates the expression of the inducible FLP or modifies the accessibility of the FRT flanking sequences, resulting in some transformants with non-functional FLP machinery. Previous work also shows that both expression of heterogeneous genes [54] and function of FRT/FLP can depend on the genomic context. This suggests that some genes, *URA3* for example, may not be amenable to this particular molecular genetic approach, but that other locations in the genome might be more effectively targeted for marker recycling [26,55].

Here we have identified a reasonable starting point for HR-based genetic modification in *M. pulcherrima*, but we find large variation between strains and loci. This suggests that development for any application will require careful consideration of the limitations of the approach, for example expecting a need to screen high number of transformants. It also suggests that for functional genetic dissection of novel yeasts, it may be critical to find model strains of non-model yeast species from which to study core metabolic and genetic differences with the most accessible tools.

## Figures and Tables

**Table 1:** Strains used for homologous recombination.

Strain ID	Source	Substrate	Notes	Country of Origin
3047	NCYC 3047	Fruit of *Phoenix dactylifera*	Prototype strain	Egypt
2580	NCYC 2580	Unknown	20–25% lipid [48]	Unknown
FS	UBFCC 20131	Fruit of *Vitis vinifera*	Grown in raceway pond [31]	UK
4x3	NCYC 4331	Derived from 2580	Inhibitor tolerant [43,49]	UK
DH5	UBFCC 20145	Fruit of *Rubus sp.*	10–15% lipid [48]	UK
ICS48	UBFCC 201546	Fruit of *Rubus sp.*	5–10% lipid [48]	UK
ICS46	UBFCC 201548	Fruit of *Rubus sp.*	10–15% lipid [48]	UK
ICS1	UBFCC 20151	Fruit of *Rubus sp.*	Grown at scale [44]	UK
F3	UBFCC 2016F3	Derived from 2580	Formic acid tolerant [49]	UK
DH10	UBFCC 201410	Fruit of *Rubus sp.*	Growth on algae [44]	UK

**Table 2:** Primers used. Capital letters anneal to the targeted DNA whereas lowercase letters are additions to the primer. Underlined letters form a recognition site for a restriction enzyme.

Primer Name	Sequence	Tm (°C)	Elongation Time (min)
BgIIURA3upstream_FW	attaagatctGTATTCACCGATAGATAGGC	55	1
SacIIURA3upstream_RV	cataccgcggACATGGTCACTCTAGCGGGC	55	1
ApaIURA3downstream_FW	gcatgggcccTAAAAGTTGTGTTTGAGCGTCGTC	55	1
AsiSIURA3downstream_RV	tgacgcgatcgcTCAGATGAACCTCCAGAGCCA	55	1
5977HRScreen_FW	ACCTGACGTCCCGCCCATCGCGCTTTGACTACATG	55	1
NatHRScreen_RV	TCTCTCAAAGTGAAACCATCACCAGTAGC	60	1
BgIIURA3upstream_FW	gcatgggcccTAAAAGTTGTGTTTGAGCGTCGTC	60	1

**Table 3:** Homologous recombination (HR) rates of 10 different *M. pulcherrima* strains when targeting *URA3* and their survivability after the heat shock (HS) that takes place during the transformation protocol.

Strain	Colonies Screened	% Survival in Additional Nat	% Colonies with HR	%Colonies with HR Surviving	Mean % Survived and HS	SD of Colonies Surviving HS
3047	100	36	1	2.77	11,67	6.66
2580	100	16	2	12.5	42	5.20
FS	100	16	0	0	26.33	19.04
4x3	100	16	0	0	19.67	1.53
DH5	100	13	0	0	29.67	7.57
ICS48	100	11	0	0	63.33	13.80
ICS46	100	10	0	0	34.33	5.86
ICS1	100	8	0	0	40.33	9.29
F3	30	33.66	0	0	19.67	15.57
DH10	20	20	0	0	17.67	6.03
